# Carbon nanotube integrated MOF-derived ZnCo_2_O_4_: a nanohybrid electrochemical platform for riboflavin sensing

**DOI:** 10.1039/d6ra00420b

**Published:** 2026-03-26

**Authors:** Ankita K. Dhukate, Sajid B. Mullani, Navaj B. Mullani, Tukaram D. Dongale, Sagar D. Delekar

**Affiliations:** a Department of Chemistry, Shivaji University Kolhapur Maharashtra 416004 India sddelekar7@rediffmail.com +91-231-2692333 +91-231-2609100; b School of Nanoscience and Biotechnology, Shivaji University Kolhapur Maharashtra 416004 India; c School of Physics, Center for Research on Adaptive Nanostructures and Nanodevices, Advanced Material and Bioengineering Research Centers Trinity College Dublin Ireland; d Department of Chemistry Bhogawati Mahavidyalaya, Kurukali Maharashtra 416001 India

## Abstract

Metal–organic framework (MOF) derived spinel metal oxides have attracted significant interest as electrochemical transducers due to their high surface area, rich redox-active sites, and tunable porous architectures. A nanohybrid sensing platform based on MOF-derived ZnCo_2_O_4_, integrated with multi-walled carbon nanotubes (MWCNT) was developed to enhance electrical conductivity and electron-transfer kinetics for riboflavin (RF) sensing. Structural and physicochemical analyses confirmed the formation of a crystalline spinel ZnCo_2_O_4_ framework uniformly decorated with MWCNT. XRD confirmed the formation of a single-phase spinel ZnCo_2_O_4_ with well-defined diffraction peaks, validating the successful MOF to oxide transformation, while BET indicated a high surface area of 198.32 m^2^ g^−1^. FTIR/Raman verified metal-oxide lattice bonding, whereas the electrochemical impedance spectroscopy demonstrated the significantly reduced charge-transfer resistance (*R*_ct_ = 104.4 Ω), indicative of enhanced conductivity using active-site of the nanocomposite. Electrochemical characterization revealed that the ZnCo_2_O_4_/MWCNT/GCE exhibited significantly higher current response. The sensor displayed a linear detection range of 0.01–1.2 µM (or 10–1200 nM) and a low detection limit of 0.2615 nM, as determined from DPV calibration. Kinetic analysis confirmed a diffusion-controlled electrochemical process of RF on ZnCo_2_O_4_/MWCNT/GCE, with dynamic electron-transfer behaviour. The electrode also demonstrated excellent repeatability (RSD = 1.41%, *n* = 4) and storage stability (signal loss = 5.85% over 8 days). The designed sensor was successfully applied to pharmaceutical tablet samples using the standard addition method, yielding recovery values of 93–94%, highlighting its robustness and suitability for precise RF quantification in complex matrices. These results confirm that carbon-nanotube integration significantly reinforces the electrocatalytic activity of MOF-derived ZnCo_2_O_4_, establishing the ZnCo_2_O_4_/MWCNT nanohybrid as a highly sensitive, accurate and practical electrochemical platform for RF determination, demonstrates the benefits of CNTs integration on MOF-derived spinel electrodes.

## Abstract 2

Electrochemical detection of riboflavin (vitamin B_2_) on ZnCo_2_O_4_/MWCNT modified GCE.

## Graphical abstract

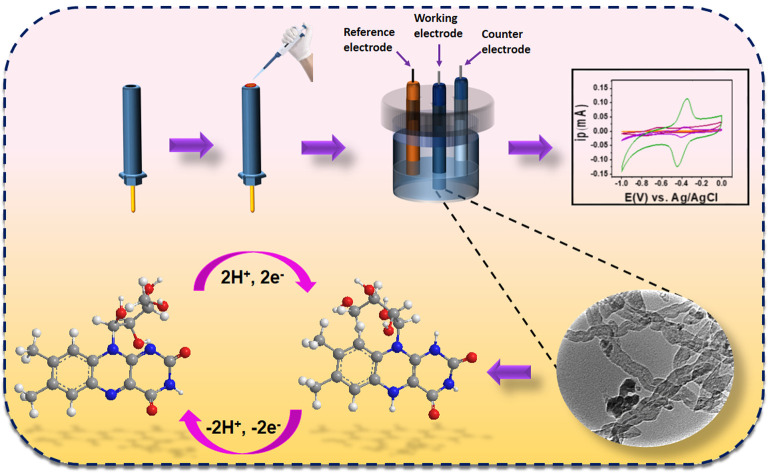

## 1. Introduction

Riboflavin (7,8-dimethyl-10-ribityl-isoalloxazine, RF) is known as vitamin B_2_ and was recognized in milk in 1979. RF is crucial for digestion and biochemical responses in humans.^1^ RF is not produced in sufficient quantities by the human body; although gut bacteria can synthesize small amounts, it is not sufficient to meet daily requirements. Therefore, it must be obtained from dietary sources, such as vegetables, mushrooms, eggs, dairy products, nuts, seeds, meat, and fruits.^2^ RF deficiency can cause disorders of the eyes, skin, and nervous system, as it plays a vital role as a coenzyme in metabolic pathways essential for energy production and cellular function.^3^ Because of its importance, it is critical to develop reliable strategies for riboflavin assessment to ensure adequate intake and prevent deficiency-related disorders.^4^ Fluorescence, chromatographic, spectroscopic, fluorometric, and electrochemical analyses of RF have been reported previously.^5^ Among the various analytical approaches, electrochemical techniques are widely recognized for the detection of biologically important molecules.^6^ Electrochemical techniques, such as cyclic voltammetry (CV), are increasingly favoured for the determination of RF due to their simple operation, rapid analysis, minimal consumption of analyte and solvent, and high sensitivity and selectivity.^7^ Compared with classical methods (as spectrophotometry or chromatography), electrochemical procedures are considered quick, simple, sensitive, and cost-effective alternatives for the analysis of biologically significant compounds.^8^ Chemically modified fabricated sensors offer significant advantages, including a larger surface area with more active sites, reduced overpotential, and enhanced responsiveness, selectivity, and detection sensitivity.^9^

In recent years, nanomaterials have experienced rapid advancement, with porous materials attracting considerable research interest. Among them, metal–organic frameworks (MOFs) represent a unique class of porous materials formed through the coordination interactions between metal ions and multifunctional organic ligands, resulting in well-defined and highly ordered structures.^10^ The tunable structural and compositional features of MOFs make them promising candidates for adsorption, proton conduction, and electrode materials across electrochemical sensing, supercapacitor, and water-splitting applications.^11^ Zeolitic imidazolate frameworks (ZIFs) are a subclass of MOFs formed *via* the self-assembly of metal ions and imidazolate linkers. They have attracted considerable research interest due to their high surface area, exceptional porosity, well-defined crystallinity, and robust structural stability. As a result, ZIF materials demonstrate significant potential for applications in electrochemical sensing.^12^ ZIF-8 has several distinctive features, including a sodalite-type framework structure, a high specific surface area, and exceptionally high porosity.^13^ Zeolitic imidazolate framework-67 (ZIF-67) is a cobalt-based metal–organic framework formed by the coordination of 2-methylimidazole ligands with Co^2+^ ions. ZIF-67 possesses several advantageous features, including diverse compositional possibilities, high surface area, excellent porosity, abundant metal content, good chemical stability, and a tunable structural architecture.^14^ In recent years, ZIF materials have been extensively utilized as precursors for the synthesis of metal oxide/carbon composite materials, as the metal centers present in ZIF structures can be transformed into metal oxides during the calcination process,^15^ These can be further integrated with carbon-based materials, such as carbon nanotubes (CNTs), thus enhancing the stability and improving the electrical conductivity of the resulting composites.^16^ Lin *et al.* initiated research utilizing metal–organic frameworks (MOFs) as versatile platforms for integrating molecular functionalities into solid-state materials.^17^ These materials offer several notable advantages, including good environmental compatibility, cost-effectiveness, and the availability of abundant natural resources. In particular, ZnCo_2_O_4_ with a spinel crystal structure has emerged as a promising material for electrode modification in electrochemical applications. Zinc doping in MOF-derived Co_3_O_4_ forming ZnCo_2_O_4_ significantly enhances glucose sensing performance through increased conductivity and active sites, as demonstrated by Divyarani *et al.* who achieved 24.8 nM LOD using ZnCo_2_O_4_@MOF composite.^18^

Integrating MWCNTs with MOF-derived metal oxides enhances RF sensing by improving electrical conductivity, analyte adsorption, and electron transfer, resulting in higher sensitivity.^19^ A Mn_3_Co_3_O_4_/MWCNT nanocomposite-modified SPCE was reported for furazolidone detection, where the introduction of MWCNTs markedly improved the electrochemical response by facilitating faster electron transfer and increased active surface area. The sensor exhibited an ultralow detection limit of 0.55 nM and a wide linear range of 0.05–650 µM, along with good selectivity, reproducibility, and storage stability.^20^ Although MOF-derived metal oxides have been extensively investigated for glucose and antibiotic sensing, their application toward riboflavin detection remains largely unexplored. Riboflavin exhibits complex redox behavior and requires highly efficient electron transfer kinetics for sensitive detection. The spinel structure of ZnCo_2_O_4_ has Co^2+^/Co^3+^ sites, facilitates proton-coupled electron transfer critical for RF oxidation, MOF-derived porous architecture maximizes surface area and catalytic site exposure, while MWCNT integration enhances conductivity and minimizes charge-transfer resistance. In this work, the integration of MOF-derived ZnCo_2_O_4_ with conductive MWCNT provides a synergistic platform combining high electroactive surface area (2.34 cm^2^) and reduced charge-transfer resistance (104.4 Ω). This structural and electronic optimization enables ultrasensitive detection of RF with a remarkably low detection limit of 0.2615 nM and a wide linear range of 10–200 nM. Compared to previously reported MOF-derived metal oxide sensors, the present system demonstrates enhanced catalytic efficiency and improved analytical performance specifically for RF sensing.^21^

## 2. Experimental section

### 2.1 Materials

All reagents used in this study were of analytical grade and were used as received without further purification. The chemicals were obtained from the Merck.

### 2.2 Acid functionalization of MWCNT

0.5 g of multi-walled carbon nanotubes (MWCNT) were dispersed in 75 mL of H_2_SO_4_, 25 mL of HNO_3_ and 25 mL of distilled water. The MWCNT were then refluxed for 6 h at 110 °C to facilitate the breakdown of CNT agglomerates and to ensure that the acid solution penetrated the CNT surfaces. After the reaction, the solution was allowed to cool to room temperature, with the aid of an ice bath to accelerate the cooling. To remove excess acid and neutralize the solution, the MWCNT was washed with distilled water several times (approximately to 5–6 washes) until the pH of the filtrate became neutral. Filtration was performed using a Buchner funnel and filter paper to separate the functionalized MWCNT from the liquid. The filtered MWCNT were then dried in an oven at 60 °C for 12–24 h to remove any residual moisture.

### 2.3 Synthesis of ZnCo-ZIF MOF

A mixture of 297 mg Zn(NO_3_)_2_·6H_2_O and 582 mg Co(NO_3_)_2_·6H_2_O was dissolved in 30 mL of methanol under constant stirring to obtain a homogeneous solution. In a separate beaker, 984 mg of 2-methylimidazole was dissolved in 10 mL of methanol to prepare a clear solution. The two solutions were subsequently combined and left undisturbed for 24 h to allow the reaction to proceed. The resulting purple precipitate was collected by centrifugation, washed repeatedly with ethanol three times, and subsequently dried at 60 °C for 12 h.

### 2.4 Synthesis of Co_3_O_4_ and ZnCo_2_O_4_

The Co_3_O_4_ and ZnCo_2_O_4_/MWCNTs were prepared by directly calcining the above synthesized ZIF-67 and ZnCo-ZIF MOF NC under a nitrogen at 900 °C for 2 h with a controlled heating rate of 3 °C min^−1^. The obtained black powders were subsequently subjected to annealing in air at 250 °C for 2 h with a heating rate of 2 °C min^−1^, resulting in the formation of Co_3_O_4_ and ZnCo_2_O_4_.

### 2.5 Synthesis of ZnCo_2_O_4_/MWCNT NC

ZnCo-ZIF MOF was dispersed in 50 mL of distilled water under continuous stirring. Subsequently, a 5 wt% suspension of acid-treated MWCNT in water was introduced, and the mixture was stirred for 60 min to obtain a uniform dispersion. Finally, the obtained material was separated by centrifugation and then subjected to calcination under a nitrogen atmosphere at 900 °C for 2 h, employing a controlled heating rate of 3 °C min^−1^. Subsequently, the obtained black powders were annealed in air at 250 °C for 2 h with a heating rate of 2 °C min^−1^, resulting in the formation of the ZnCo_2_O_4_/MWCNT NC.

### 2.6 Materials characterizations

The structural properties of the synthesized materials were examined using X-ray diffraction (XRD) with a Bruker AXS D8 Advance diffractometer employing Cu Kα radiation (*λ* = 1.5406 Å). Functional groups were identified by Fourier transform infrared spectroscopy (FTIR) using a Bruker Alpha FTIR spectrometer in the range of 4000–400 cm^−1^. Raman spectra were recorded using a Bruker Multi-RAM spectrometer with a 532 nm excitation laser. The morphology and particle size were analyzed by high resolution transmission electron microscopy (HRTEM) using a JEOL JEM 2100 Plus transmission electron microscope. Surface elemental composition and chemical states were investigated through X-ray photoelectron spectroscopy (XPS) using instruments such as the VG Multilab 2000 XPS and Thermo Scientific K-Alpha XPS spectrometer. The surface area and pore characteristics were determined from N_2_ adsorption–desorption measurements using the BET method on a Quantachrome NOVA1000e surface area analyzer.

### 2.7 Preparation of working electrode

Before surface modification, the glassy carbon electrode (GCE) was treated with a diluted nitric acid solution (1 mL HNO_3_ in 9 mL DDW) to remove surface impurities. For the preparation of modified electrodes, nanomaterial dispersions were prepared by dispersing 10 mg of Co_3_O_4_, ZnCo_2_O_4_, and ZnCo_2_O_4_/MWCNT nanocomposites in 1 mL of an ethanol/DDW mixture (1 : 1, v/v), followed by ultrasonication to prepare a stable and uniform dispersion. The electrode surface was then modified through a drop-casting method by depositing 20 µL nanomaterial suspension onto the cleaned GCE using a micropipette. Subsequently, the modified electrodes were allowed to dry at room temperature overnight to ensure complete solvent evaporation and firm immobilization of the active materials on the electrode surface. The fabricated electrodes were then employed for subsequent electrochemical investigations.

### 2.8 Electrochemical analysis

Electrochemical measurements were carried out using a CH Instruments D650 potentiostat. Differential pulse voltammetry (DPV), cyclic voltammetry (CV), and electrochemical impedance spectroscopy (EIS) were employed to evaluate the electrochemical properties of the modified electrodes. All measurements were carried out using a conventional three-electrode configuration, comprising a modified glassy carbon electrode (GCE) as the working electrode, a platinum wire as the counter electrode, and an Ag/AgCl (saturated KCl) electrode as the reference electrode. The study used a 0.1 M phosphate buffer solution (PBS, pH 7) prepared in DDW as the supporting electrolyte.

## 3. Result and discussion

### 3.1 XRD studies

**Fig. 1:**
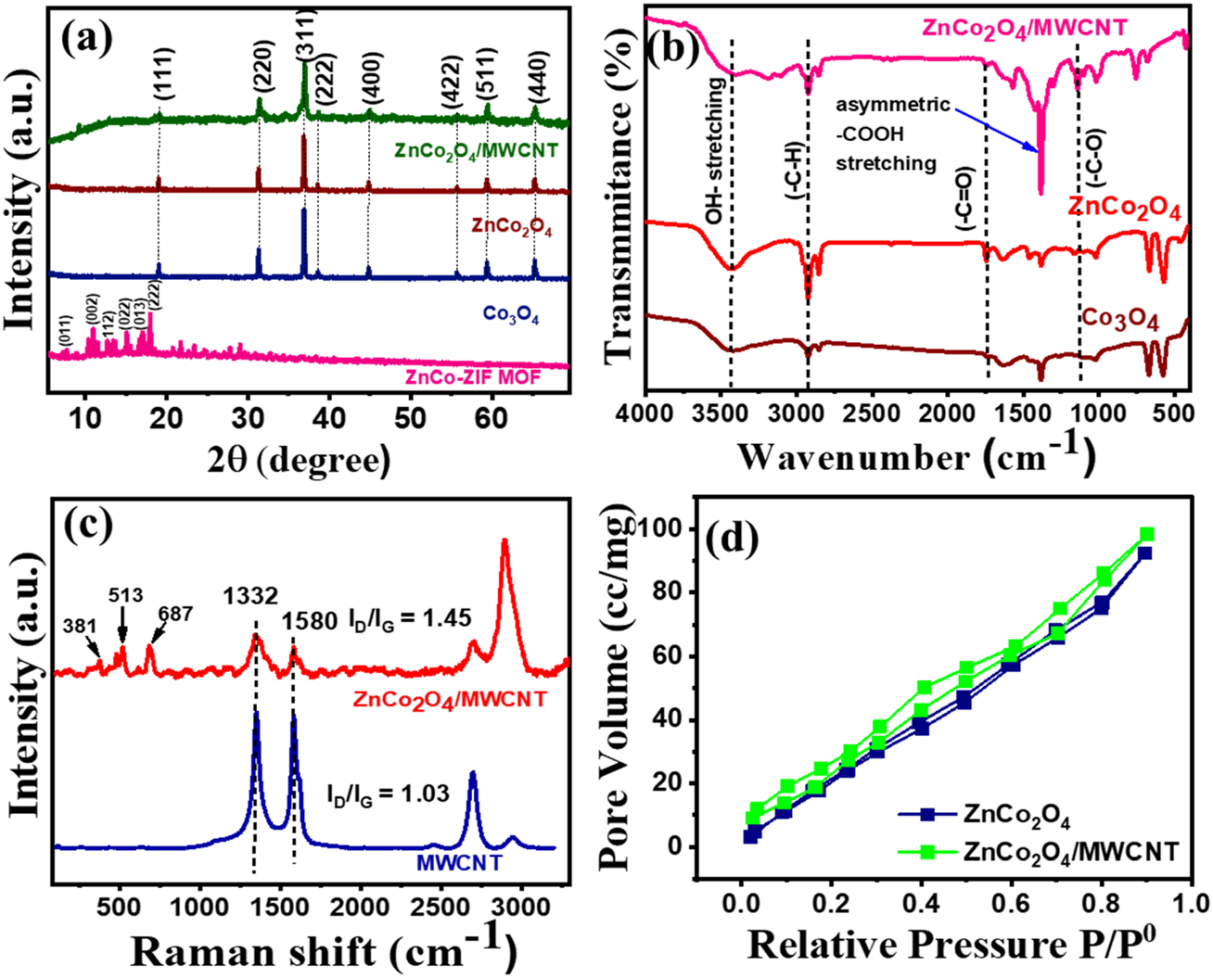
(a) XRD patterns of CoZn-ZIF MOF, Co_3_O_4_, ZnCo_2_O_4_, ZnCo_2_O_4_/MWCNT, (b) FTIR spectra of Co_3_O_4_, ZnCo_2_O_4_, ZnCo_2_O_4_/MWCNT, (c) Raman spectra of MWCNT, ZnCo_2_O_4_/MWCNT, (d) N_2_ adsorption–desorption isotherms of the ZnCo_2_O_4,_ ZnCo_2_O_4_/MWCNT.

The XRD patterns of Co_3_O_4_, ZnCo_2_O_4_, ZnCo_2_O_4_/MWCNT, and ZnCo-ZIF MOF are displayed in Fig. 1a, where the peak with 2*θ* values at ∼26° corresponds to the (002) plane of hexagonal graphite, representing the interlayer spacing between the concentric graphene walls in the nanotubes of the MWCNTs (Fig. S1). The XRD pattern of ZnCo-ZIF MOF is shown in Fig. 1a, distinct diffraction peaks are detected at 2*θ* values of 7.4°, 10.4°, 12.8°, 14.7°, 16.5°, and 18.1°, corresponding to the (011), (002), (112), (022), (013), and (222) lattice planes, respectively.^22^ For the ZnCo_2_O_4_/MWCNT (Fig. 1a), the characteristic diffraction peaks located at 2*θ* values of 30.95°, 36.96°, 38.77°, 44.18°, 55.48°, 59.49°, and 64.71° corresponding to the (220), (311), (222), (400), (422), (511), and (440) crystal planes respectively. The observed diffraction peaks are consistent with the standard XRD pattern of cubic ZnCo_2_O_4_ (JCPDS 023-1390/4-0117).^23^ The ZnCo_2_O_4_ pattern exhibit peaks at the same 2*θ* positions and similar Miller indices as Co_3_O_4_, with no separate ZnO or Co_2_O_3_ impurity peaks, indicating the formation of pure Co_3_O_4_ and ZnCo_2_O_4_ phases.^24^ A reduction in intensity is observed in the XRD pattern of ZnCo_2_O_4_/MWCNT, suggesting a low crystallite size compared to Co_3_O_4_ and ZnCo_2_O_4_, as well as the dilution of oxide by the carbon matrix. The introduction of MWCNT into ZnCo_2_O_4_ has no significant effect on the structure of ZnCo_2_O_4_/MWCNT,^25^ The Crystallite/grain sizes of ZnCo_2_O_4_ and ZnCo_2_O_4_/MWCNT were calculated by using the Debye–Scherrer equation to be 18.6 nm and 29.2 nm, respectively.

### 3.2 FTIR analysis

The Fourier transform infrared (FTIR) spectra of Co_3_O_4_, ZnCo_2_O_4_ and ZnCo_2_O_4_/MWCNT (Fig. 1b) show broad bands at ∼3438 and ∼1633 cm^−1^ assigned to the O–H stretching and bending vibrational modes,^26^ respectively. The other bands at ∼1630 and ∼2920 cm^−1^ corresponds to C=C stretching vibrations and C–H stretching frequency of MWCNT,^27^ respectively. In addition, the FTIR spectra showed major bands at 500–700 cm^−1^, indicating metal oxygen vibrational modes. In the case of ZnCo_2_O_4_/MWCNT, the intensities of the characteristic peaks corresponding to the functional groups are slightly diminished, suggesting a reduced surface presence of ZnCo_2_O_4_ due to the incorporation of MWCNT. The peak intensity in of the O–H stretching in ZnCo_2_O_4_/MWCNT is diminished due to the formation of an ester bond between the (–OH) group present on the metal oxide and the (–COOH) group of the MWCNT, along with an increase in the intensity of the C–O stretching frequency ∼1100 cm^−1^.^28^

### 3.3 Raman analysis

The interactions and structural integrity of the MWCNT and ZnCo_2_O_4_/MWCNT were characterized using Raman spectroscopy (Fig. 1c). The Raman peaks located at 1352 and 1583 cm^−1^ are assigned to the D and G bands, respectively. The D band is associated with defect-induced disordered vibrations in the carbon framework, whereas the G band arises from the in-plane vibration of sp^2^-hybridized carbon atoms. Additionally, the bands appearing at 381, 513, and 687 cm^−1^ are assigned to the E_g_, F_2g_, and A_1g_ vibrational modes of ZnCo_2_O_4_, respectively.^29^ In general, the ratio of the intensities of the D and G bands (*I*_D_/*I*_G_) is commonly used as an indicator of the degree of structural disorder in carbon-based materials.^30^ In this study, the intensity ratios (*I*_D_/*I*_G_) were determined to be 1.45 for the ZnCo_2_O_4_/MWCNT nanocomposite and 1.03 for pristine MWCNT.^31^

### 3.4 BET studies

The N_2_ adsorption isotherms of ZnCo_2_O_4_ and ZnCo_2_O_4_/MWCNT exhibited a characteristic type IV isotherm, indicating the mesoporous nature of these materials.^32^ The isotherm exhibits a gradual increase in the adsorbed pore volume with increasing relative pressure (*P*/*P*_0_ = 0–0.9) is observed, which indicates multilayer adsorption followed by capillary condensation in the mesoporous.^33^ The surface area of ZnCo_2_O_4_/MWCNT (198.32 m^2^ g^−1^) was higher than that of ZnCo_2_O_4_ (123.58 m^2^ g^−1^). As shown in Fig. 1d ZnCo_2_O_4_/MWCNT composite displays a relatively higher pore volume over the entire pressure range compared to pristine ZnCo_2_O_4_, suggesting that the incorporation of MWCNT generates additional mesoporous channels and inhibits nanoparticle aggregation. The enhanced pore volume and mesoporous architecture are expected to facilitate electrolyte penetration and improve mass transport, which are advantageous for the electrochemical sensing of RF.^34^

### 3.5 Thermal analysis

**Fig. 2:**
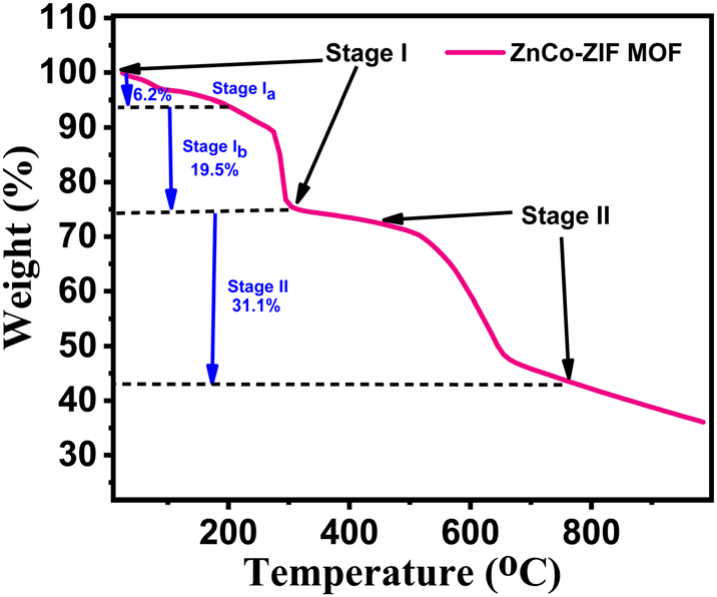
Thermogravimetric analysis of ZnCo-ZIF MOF.

Fig. 2 shows the thermogram of ZnCo-ZIF MOF, recorded under nitrogen atmosphere from room temperature to 900 °C, which shows a multistep weight-loss process over the temperature range of room temperature to 900 °C, indicating the sequential removal of physically adsorbed species and decomposition of the organic framework. The initial weight loss up to a temperature of 160 °C is attributed to the loss of lattice water present in the template and further loss up to 310 °C for loosely coordinated species within the pore channels of the MOF. According to reported studies, the removal of lattice water from such templates typically occurs at temperatures up to about 150 °C. The thermogravimetric analysis reveals that the initial weight loss of 6.2% (stage *I*_a_) corresponds to the removal of lattice water, while the subsequent weight reduction of approximately 12.93% (stage *I*_b_) is attributed to the elimination of DMF from the as-synthesized template. A relatively stable region with minimal mass change (less than 6.0%) is observed between 205 and 410 °C, indicating the thermal stability of the template within this temperature range. Above 410 °C, a significant weight loss of 43.19% occurs, which can be ascribed to the decomposition of the organic ligands (2-methylimidazole) present in the MOF structure. The complete decomposition of the organic components up to 600 °C suggests the formation of a stable ZnCo_2_O_4_ composite.^35^

### 3.6 Morphology analysis

**Fig. 3:**
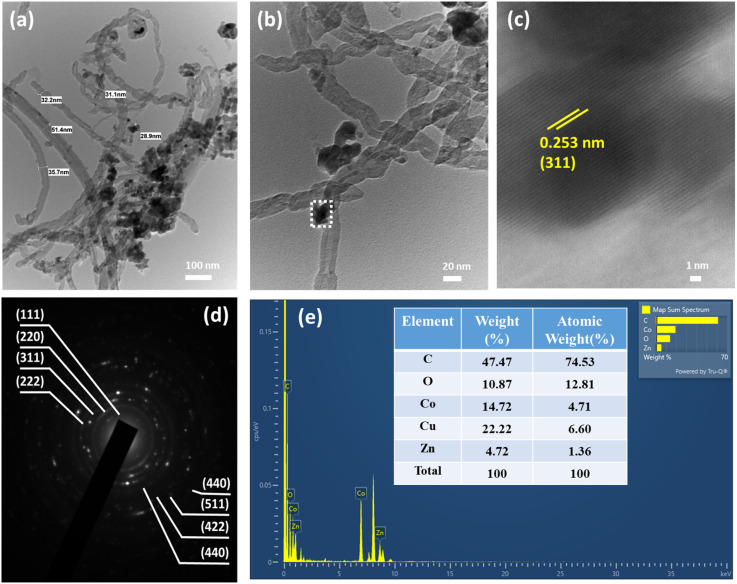
TEM images of (a and b) ZnCo_2_O_4_/MWCNT, (c) HRTEM of ZnCo_2_O_4_/MWCNT, (d) SAED pattern of ZnCo_2_O_4_/MWCNT and (e) EDX spectra of ZnCo_2_O_4_/MWCNT.

The structural morphology, microstructure, and average size of the as-synthesized ZnCo_2_O_4_/MWCNT NC were investigated using high-resolution transmission electron microscopy (HRTEM). HRTEM was used to further examine the structure of the synthesized ZnCo_2_O_4_/MWCNT nanocomposites. The images (Fig. 3a and b) show that the ZnCo_2_O_4_ nanoparticles were well dispersed along the outer surface of the carboxyl-functionalized MWCNT. In the micrographs, the nanoparticles appear as dark spots, whereas the nanotube framework appears lighter, making the contrast between the two phases clear. The average diameters of the MWCNT and ZnCo_2_O_4_ nanoparticles were about 33.9 ± 1.7 nm and 30.0 ± 1.1 nm, respectively. Most nanoparticles were found to fall within a narrow size range of 28–32 nm, indicating good uniformity in the nanocomposite.^36^

The selected area electron diffraction (SAED) patterns for the synthesized ZnCo_2_O_4_/MWCNT NC consisted of typical polycrystalline diffraction rings, suggesting a nanocrystalline structure (Fig. 3d). As shown in Fig. 3c, the ZnCo_2_O_4_ nanoparticles display a distinct octahedral morphology with clearly visible lattice fringes. The measured interplanar spacing of approximately 0.253 nm can be assigned to the (311) crystal plane, which is in excellent agreement with the standard JCPDS data (no. 23-1390) referenced in the XRD analysis. This agreement between the HRTEM and XRD results further supports the successful formation of the spinel ZnCo_2_O_4_ structure. In addition, the MWCNT exhibited several defect sites arising from the presence of carboxyl functional groups, which facilitate the effective anchoring of ZnCo_2_O_4_ nanoparticles. As illustrated in Fig. 3b, ZnCo_2_O_4_ nanoparticles are uniformly distributed and closely attached along the surface of the MWCNT. The elemental composition and distribution of the ZnCo_2_O_4_/MWCNT composite were further examined using EDX analysis. The elemental mapping images demonstrate a homogeneous distribution of C, O, Zn, and Co throughout the composite (Fig. S2). Moreover, the EDX spectrum (Fig. 3e) displays prominent peaks corresponding to C, O, Zn, and Co with atomic percentages of 74.53%, 12.81%, 1.36%, and 4.71%, respectively. Ideally, the atomic ratio of Zn, Co, and O in ZnCo_2_O_4_ is 1 : 2 : 4. However, the incorporation of MWCNT increases the relative oxygen content while reducing the proportions of Zn and Co. The high carbon content originating from MWCNT significantly improves the electrical conductivity of the composite material.

### 3.7 XPS studies

**Fig. 4:**
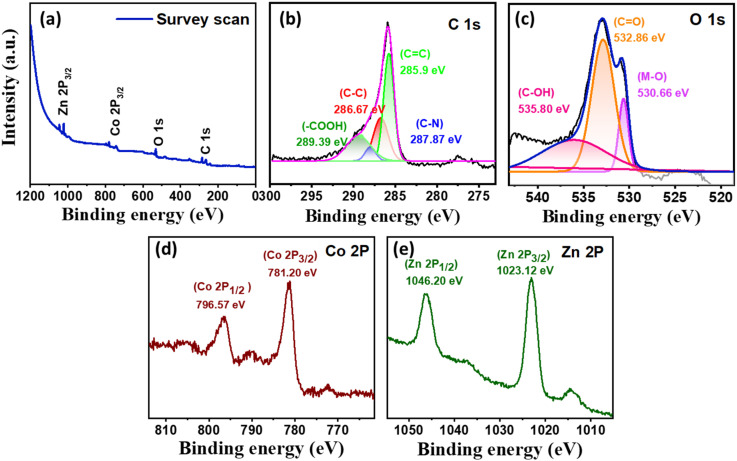
(a) XPS survey spectra of ZnCo_2_O_4_/MWCNT and deconvoluted XPS spectrum of (b) C 1s, (c) O 1s, (d) Co 2p (e) Zn 2p.

The XPS analysis of the ZnCo_2_O_4_/MWCNT NC is presented in Fig. 4. The survey spectrum (Fig. 4a) indicates the presence of C, N, O, Co, and Zn elements within the composite material. As shown in Fig. 4b, the C 1s spectrum exhibits a peak at 285.9 eV corresponding to sp^2^-hybridized graphitic carbon, while the peak at 286.7 eV is attributed to sp^3^-hybridized carbon. Furthermore, the peaks located at 287.9 eV and 289.4 eV are associated with C–O/C–N functional groups and carboxyl (–COOH) groups, respectively. The O 1 s spectrum (Fig. 4c) exhibits a peak at 530.7 eV, which is attributed to metal–oxygen bonds, whereas the peaks located at 532.9 eV and 535.8 eV correspond to C=O and C–O functional groups on the MWCNT surface. The high-resolution Co 2p spectrum exhibits two prominent peaks at 781.2 eV (Co 2p_3/2_) and 796.5 eV (Co 2p_1/2_), with a spin–orbit splitting of Δ*E* = 15.28 eV. These values align with reported data for Co^3+^ in spinel ZnCo_2_O_4_,^37^ confirming the dominance of the Co^3+^ oxidation state in ZnCo_2_O_4_/MWCNT NC. The high-resolution Zn 2p spectrum is presented in Fig. 4e, Two peaks observed at 1046.2 and 1023.1 eV are ascribed to Zn 2p_1/2_ and Zn 2p_3/2_. The observed binding energies and the spin–orbit separation (23.15) are consistent with Zn^2+^ species, confirming the presence of zinc in its oxidized state within the spinel ZnCo_2_O_4_ structure.^38^

### 3.8 Impedance and electrochemical performance evaluation

**Fig. 5:**
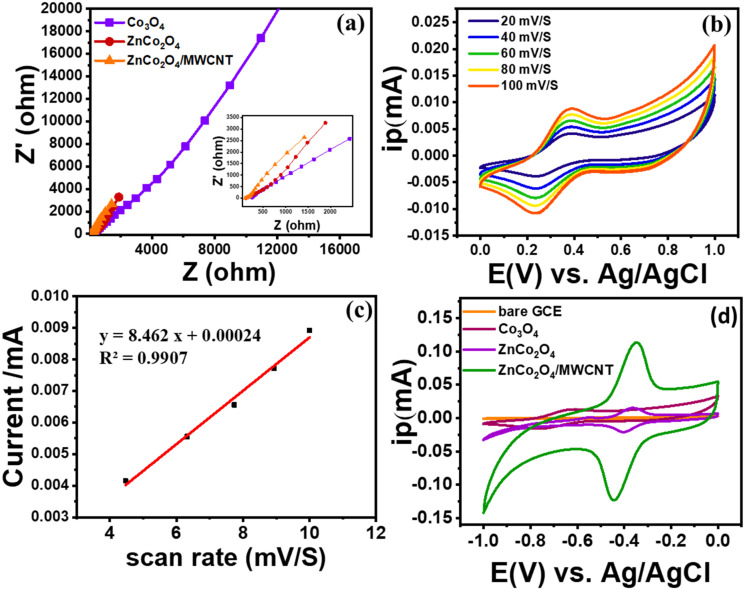
(a) Nyquist plots obtained for the modified electrodes (Co_3_O_4_/GCE, ZnCo_2_O_4_/GCE, and ZnCo_2_O_4_/MWCNT/GCE), (b) CV profiles of ZnCo_2_O_4_/MWCNT/GCE at different scan rates in 0.5 mM K_4_ [Fe (CN)_6_], (c) fitted relationship between *I*_pa_ and *I*_pc_ w.r.t. scan rate, (d) CVs of bare GCE, Co_3_O_4_/GCE, ZnCo_2_O_4_/GCE, ZnCo_2_O_4_/MWCNT/GCE in 0.1 M PBS (pH 7) for 1 µM RF concentration.

To understand the electron transfer characteristics of the modified electrodes (Co_3_O_4_/GCE, ZnCo_2_O_4_/GCE, and ZnCo_2_O_4_/MWCNT/GCE), electrochemical characterization was performed by electrochemical impedance spectroscopy (EIS) and cyclic voltammetry (CV) in 5 mM K_4_[Fe (CN)_6_] prepared in 0.1 M KCl. EIS was employed to elucidate the charge-transfer kinetics and interfacial resistance of the synthesized electrodes. The Nyquist plots (Fig. 5a) show a depressed semicircle in the high-medium frequency region and a sloped linear segment at low frequencies, indicating the charge-transfer resistance (*R*_ct_) for Co_3_O_4_, ZnCo_2_O_4_, and ZnCo_2_O_4_/MWCNT electrodes respectively. Among the three materials, the ZnCo_2_O_4_/MWCNT electrode demonstrated the smallest semicircle diameter, indicative of the lowest *R*_ct_ (104.4 Ω) and the most efficient charge-transfer process. This improvement was attributed to the highly conductive MWCNT framework, which provides rapid electron transport pathways and enhanced interfacial contact with the electrolyte. In comparison, Co_3_O_4_ exhibited the largest *R*_ct_ (406 Ω) value, suggesting sluggish electron/ion transfer, while ZnCo_2_O_4_ showed an intermediate *R*_ct_ (280 Ω). The inset of Fig. 5a shows the low-impedance region, further confirming the reduced resistance of ZnCo_2_O_4_/MWCNT NC.

Following the EIS results indicating enhanced charge transfer and lower interfacial resistance at the ZnCo_2_O_4_/MWCNT/GCE, it was necessary to evaluate the electroactive surface area (ESA) to quantify the extent of active sites contributing to the electrochemical response. Cyclic voltammograms recorded at different scan rates in the 0.5 mM K_4_[Fe (CN)_6_] redox system (Fig. 5b) showed a progressive increase in peak current with increasing scan rate. The ESA of each modified electrode was determined using the Randles–Sevcik eqn (A), where the ESA was calculated from the slope of the linear *I*_p_*vs. ν*^1/2^ plot (Fig. 5c), indicating a diffusion-controlled electron-transfer process.^39^

A *I*_pa_ = (2.69 × 10^5^) *n*^3/2^*AD*^1/2^*Cν*^1/2^

In this equation, *I*_pa_ represents the anodic peak current, *n* denotes the number of electrons participating in the redox process, *A* corresponds to the effective electroactive surface area of the electrode (cm^2^), *D* is the diffusion coefficient of K_4_[Fe (CN)_6_] (cm^2^ s^−1^), *C* indicates the concentration of the redox species (mol cm^−3^), and *ν* represents the scan rate (V s^−1^). The slope of the *I*_p_*vs. ν*^1/2^ plot for the ZnCo_2_O_4_/MWCNT/GCE modified electrode was determined to be 8.462 (A V^−1/2^ s^1/2^). Using this value in the Randles–Sevcik equation with *D* = 7.2 × 10^−6^ cm^2^ s^−1^, at 298 K, yielded an ESA of 2.34 cm^2^, indicating a significant improvement compared to Co_3_O_4_/GCE, ZnCo_2_O_4_/GCE.

### 3.9 The electrochemical behaviour of RF at modified electrodes

The GCE was modified with the synthesized Co_3_O_4_, ZnCo_2_O_4_, and ZnCo_2_O_4_/MWCNT NC. Electrochemical response of RF were studied out in 0.1 M PBS (pH 7) and potential window of −1 to 0 V. The RF oxidation at Co_3_O_4_, ZnCo_2_O_4_, and ZnCo_2_O_4_/MWCNT NC-modified electrodes was investigated using cyclic voltammetry. Fig. 5d shows the relative overlay CV results of RF at the modified Co_3_O_4_/GCE, ZnCo_2_O_4_/GCE, and ZnCo_2_O_4_/MWCNT/GCE. The electrochemical response of the modified electrodes was evaluated using 1 µM RF solution in 0.1 M PBS (pH 7) at a scan rate of 50 mV s^−1^ within a potential range of −1 to 0 V for the ZnCo_2_O_4_/MWCNT modified GCE showed a significantly higher oxidation peak current with respect to the other modified electrodes. However, ZnCo_2_O_4_/MWCNT/GCE exhibits an oxidation peak at −0.35 V with the highest current value. This enhanced performance can be attributed to the large specific surface area (198.32 m^2^ g^−1^) of ZnCo_2_O_4_/MWCNT and the enhanced electron transfer rate. These findings indicate that the ZnCo_2_O_4_/MWCNT/GCE demonstrates superior electrochemical activity toward RF detection. This enhanced performance is consistent with the lower *R*_ct_ observed in the EIS analysis (Fig. 5a) and the large specific surface area of the ZnCo_2_O_4_/MWCNT nanocomposite.^40^

**Fig. 6:**
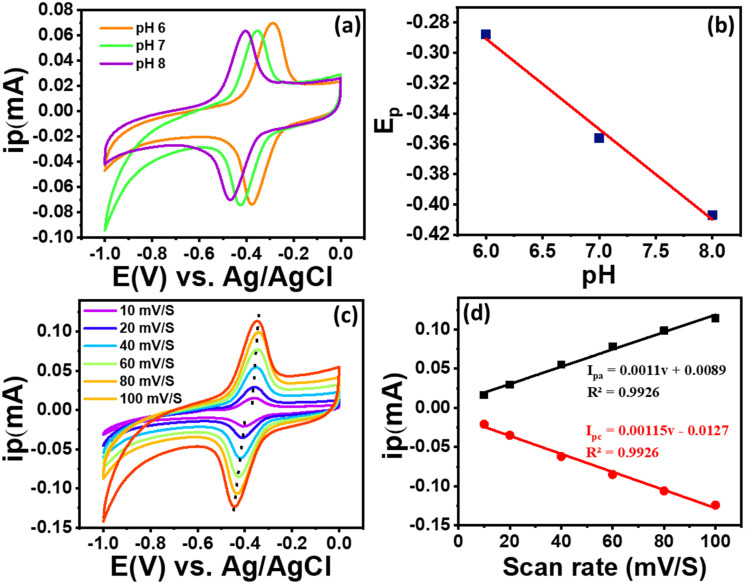
(a) CV profile of ZnCo_2_O_4_/MWCNT/GCE towards 1 µM RF at pH 6.0, 7.0 and 8.0 (0.1 M PBS), (b) fitted relationship between *E*_p_ w.r.t. pH, (c) CV profiles of ZnCo_2_O_4_/MWCNT/GCE for 1 µM RF (0.1 M PBS pH = 7) at different scan rates, (d) fitted relationship between *I*_pa_ and *I*_pc_ w.r.t. scan rate.

In electrochemical sensing, the pH of the electrolyte (PBS) plays an crucial role in electrochemical reactions.^41^ The pH variation from 6–8 (*i.e.* acidic, basic, and neutral conditions) affect the electrochemical activity of RF was investigated to optimize the pH condition of the RF sensor. Fig. 6a shows that as pH of the PBS solution increased from 6 to 8, and the value of peak potential shifted negatively, indicating that protons participated in the RF oxidation process at the modified GCE electrode surface.^42^ The slope of the *E*_p_*vs.* pH plot (Fig. 6b) (≈59.6 mV per pH) is close to the theoretical Nernstian value of 59 mV per pH, indicating that the electro-oxidation of RF at the ZnCo_2_O_4_/MWCNT-modified electrode involves an equivalent number of protons and electrons, consistent with a proton-coupled electron transfer mechanism. Laviron analysis indicates that riboflavin oxidation proceeds *via* a two-electron/two-proton process, consistent with reported electrochemical behaviour. This near-Nernstian behavior also suggests that the ZnCo_2_O_4_/MWCNT surface provides a favourable environment for RF adsorption, facilitating efficient electron-proton transfer during oxidation.^43^

The scan rate variation study of ZnCo_2_O_4_/MWCNT/GCE with RF (0.1 µM in 0.1 M PBS (pH 7)) was investigated by varying the scan rate from to 10–100 mV s^−1^ and as portrayed in Fig. 6c. It is evident that the peak current increases with an increase in the scan rate (*ν*). A linear relationship between the square root of the scan rate (*ν*^1/2^) and the peak current is observed, as illustrated in Fig. 6d. The corresponding linear regression equations are *y* = 0.0011*x* + 0.0089, *R*^2^ = 0.9947 and *y* = −0.0014*x* + 0.0068, *R*^2^ = 0.9956. The increase in peak currents with increasing scan rate indicates a diffusion-controlled electrochemical process for RF on the ZnCo_2_O_4_/MWCNT/GCE, consistent with a mass transport-limited electron transfer mechanism.^44^ Furthermore, the heterogeneous electron transfer rate constant (*k*_s_) was estimated using Laviron's model based on the peak potential separation (Δ*E*_p_ = 0.006 V) obtained at a scan rate of 10 mV s^−1^, assuming a transfer coefficient (*α*) of 0.5 and a two-electron transfer process (*n* = 2). The calculated *k*_s_ value was approximately 0.35 s^−1^, suggesting relatively fast electron transfer kinetics at the modified electrode surface.

### 3.10 Detection of RF on ZnCo_2_O_4_/MWCNT/GCE

**Fig. 7:**
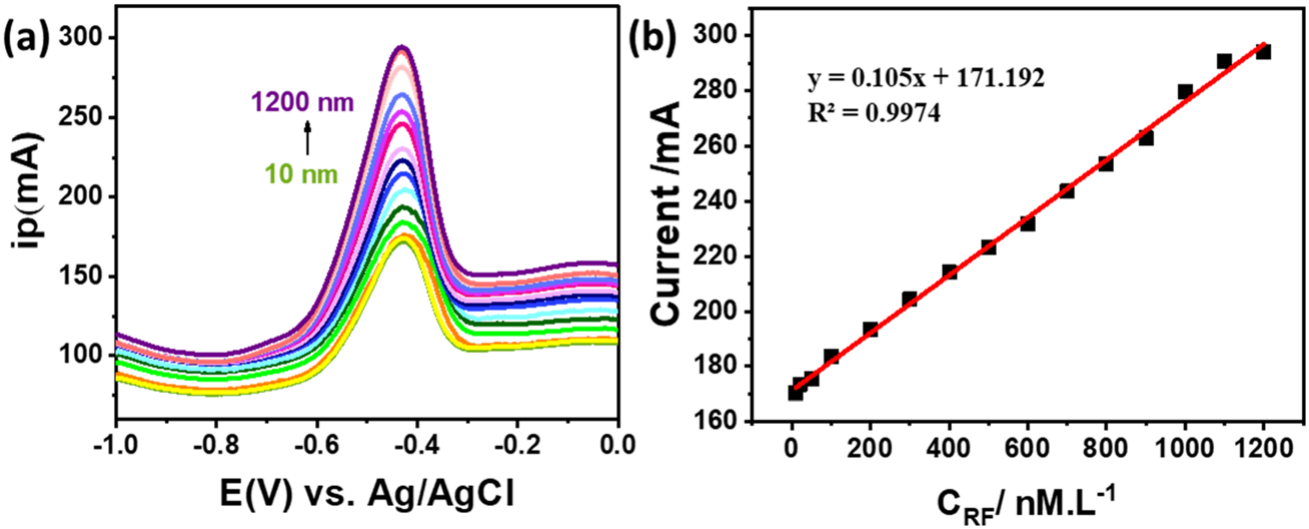
(a) DPV response of ZnCo_2_O_4_/MWCNT/GCE towards 10 to 1200 nM RF concentration in 0.1 M PBS, pH = 7, (b) the calibration curve for RF detection.

To further evaluate the detection limit and linear dynamic range of the ZnCo_2_O_4_/MWCNT/GCE, DPV was employed to examine the electrochemical response of RF at varying concentrations. Fig. 7a shows that the value of the anodic peak current rises with an increase in RF concentration (10–1200 nM) demonstrating the linear dependence of the anodic peak current (*I*_pa_) on RF concentration. The value of *I*_pa_ obtained from the linear plot in Fig. 7b was used to calculate the limit of detection (LOD) and limit of quantification (LOQ). The calibration plot exhibited a linear relationship described by the equation *I*_pa_ = (0.105*v* + 0.0172) (*R*^2^ = 0.9974) and the LOD and LOQ in 0.2 M PBS (pH 7.4) were subsequently calculated using the following equations:

B LOD = 3SD/*b*

C LOQ = 10SD/*b*

The LOD and LOQ were determined using the standard formulas eqn (B) and (C), where *σ* is the standard deviation of the blank response and *b* is the calibration curve slope. These yielded values of 0.2615 nM (LOD) and 0.7924 nM (LOQ), respectively. The sensor demonstrated excellent linearity across a broad RF concentration range (10–1200 nM). Notably, the obtained LOD values are significantly lower than those reported for previously developed electrodes. To place the present work in context, the performance characteristics of previously developed modified electrodes for RF sensing were examined (Table 1).

**Table 1:** Comparison of previously reported modified electrodes for RF detection

Modified electrode	Method	LOD	Linear range	Ref.
Fe_3_O_4_/rGO/GCE	DPV	89 nM	0.3–100 µM	45
Ag/rGO/GCE	DPV	0.6 nM	0.002–2.2 µM	46
f-MWCNT/AuNP/PGE	DPV	35.2 nM	0.5–100 µM	47
3D-KSC/COF_TFPB-Thi_	DPV	90 nM	0.13 µM	48
			0.23 mM	
gCN.CuNF|GCE	SWV	6 nM	25 nM to 100 µM	49
g-C_3_N_4_@ZnO/GCE	DPV	0.442 nM	1.0–55 µM	50
Co_3_O_4_/rGO/AuE	SWV	1.30 µM	6.54–42.19 µM	51
R-CoP/GCN/GCE	DPV	1.09 nM	0.062–3468.75 µM	52
ZnCo_2_O_4_/MWCNT/GCE	DPV	0.2615 nM	0.01–1.2 µM	This work

### 3.11 Reproducibility, repeatability and stability studies

**Fig. 8:**
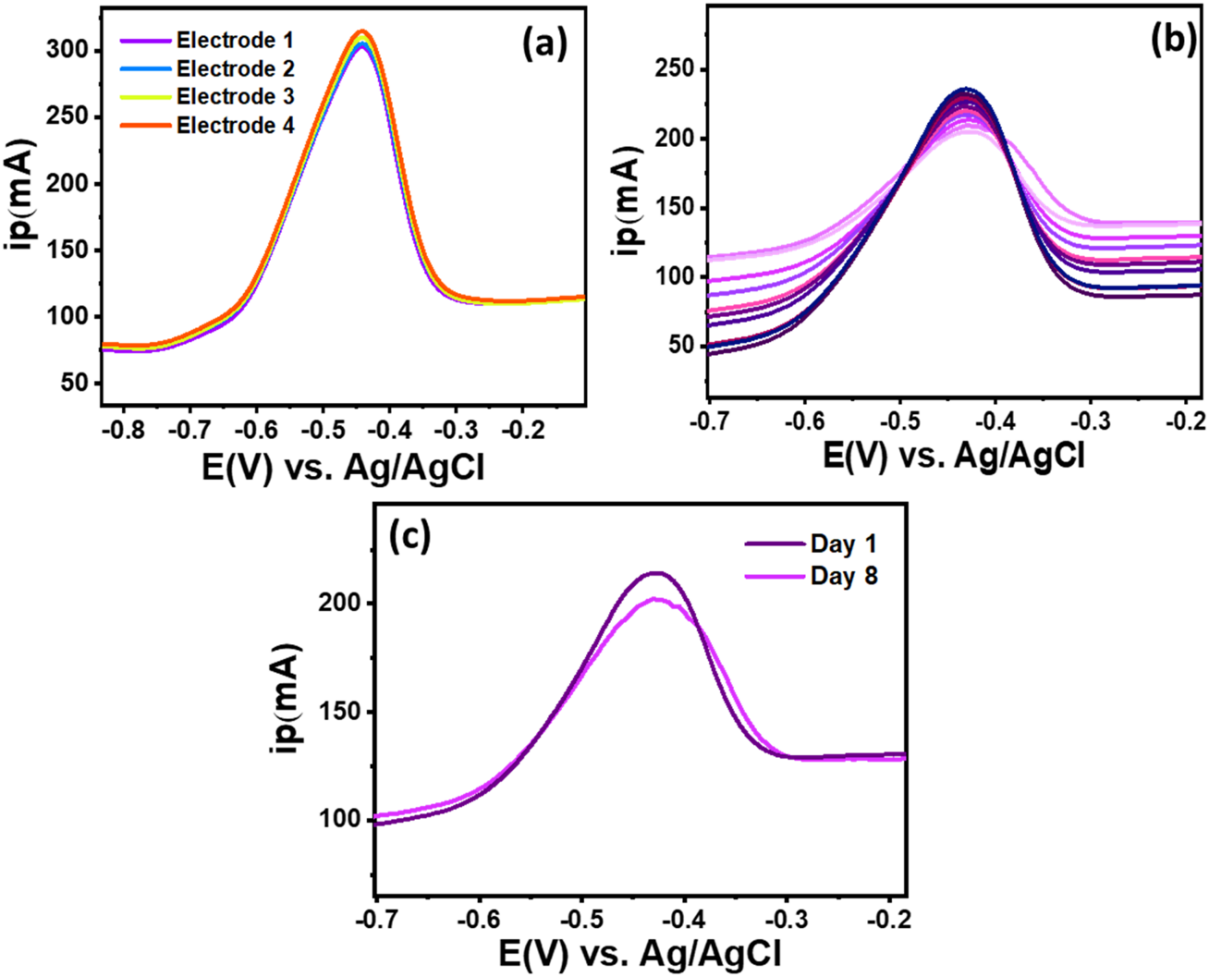
(a) Results of reproducibility studies at four different electrodes in 1 µM RF (0.1 M PBS, pH = 7), (b) DPV of the ZnCo_2_O_4_/MWCNT/GCE recorded for ten successive measurements of RF, (c) DPV responses of the ZnCo_2_O_4_/MWCNT/GCE before and after one week of storage in 0.5 µM RF(0.1 M PBS, pH = 7).

To evaluate the reproducibility of the ZnCo_2_O_4_/MWCNT/GCE for RF detection, DPV was conducted using four independently prepared electrodes with similar composition of ZnCo_2_O_4_/MWCNT/GCE; each of them were tested in a 0.5 µM (pH = 7) RF solution (Fig. 8a). All electrodes produced nearly identical anodic peak currents, with an average value of about 310 mA and a relative standard deviation (RSD) of 1.41%, demonstrating high reproducibility and minimal electrode-to-electrode variation. The ZnCo_2_O_4_/MWCNT/GCE displayed excellent repeatability with an RSD of 4.73% for ten successive measurements of RF in 50 µM (Fig. 8b). The storage stability of the ZnCo_2_O_4_/MWCNT/GCE sensor was examined by storing the electrode in a sealed container under ambient conditions for one week, and the corresponding cyclic voltammograms are shown in Fig. 8c. After storage, the sensor retained most of its electrochemical activity, exhibiting only 5.85% signal degradation for RF. Although the sensor demonstrated satisfactory stability over 8 days, extended long-term stability evaluation (2–3 weeks or longer) is required to fully establish its practical applicability, which will be addressed in future studies. These results confirm that the proposed sensor possesses good storage stability and surface robustness toward RF, supporting its potential for scalable fabrication of low-cost, portable electrochemical sensors for real-world diagnostic applications.^53^

### 3.12 Interference study

The anti-interference ability of the modified electrode toward the detection of RF was evaluated by DPV in the presence of common interfering species such as ascorbic acid (AA), uric acid (UA), thiamine (vitamin B_1_) and pyridoxine (vitamin B_6_). In this study, RF was measured in the presence of a 10-fold excess of glucose, AA, UA, glucose, vitamin B_1_ vitamin B_6_ to examine their influence on the electrochemical response. No significant change in the oxidation peak current of RF was observed after the addition of these foreign species. The results summarized in Fig. S3 indicate that the developed modified electrode exhibits excellent selectivity for RF detection even in the presence of these potentially interfering compounds with a relative standard deviation less than 2%.

### 3.13 Real-sample analysis of riboflavin in pharmaceutical tablets

The practical applicability of the ZnCo_2_O_4_/MWCNT/GCE sensor was assessed by determining RF in commercial pharmaceutical tablet samples using the standard addition method. A known quantity of finely powdered tablet was dissolved in distilled water, sonicated, and filtered to obtain a clear sample solution, which was appropriately diluted with PBS (pH 7.0) prior to analysis. The initial electrochemical response of the tablet extract was recorded, followed by the sequential addition of known concentrations of RF standard solution to the same electrolyte system. The obtained results exhibited well-defined analytical signals with satisfactory recovery values (Table 2), demonstrating the robustness, reliability and practical applicability of the developed sensor for the precise and accurate determination of RF in pharmaceutical tablet formulations.

**Table 2:** Real-time application of RF in commercial pharmaceutical tablet at ZnCo_2_O_4_/MWCNT/GCE

Added (µM)	Found (µM)	Recovery (%)	RSD (%)
10	09.40	94.0	1.32
20	18.92	94.6	1.39
30	28.12	93.7	1.24

## 4. Conclusions

In conclusion, a MOF-derived ZnCo_2_O_4_/MWCNTs nanohybrid was successfully developed as an efficient electrochemical sensing platform for RF detection. The porous ZnCo_2_O_4_ provides abundant redox-active sites, while MWCNTs ensure fast electron transport and improved surface accessibility, resulting in enhanced sensitivity and a low detection limit. The findings of the present study demonstrate that our sensor exhibited an impressively ultra-low detection limit of 0.2615 nM within a broad linear detection range of 10–1200 nM, enabling accurate and reliable measurements even at low concentrations (or at trace concentration). Additionally, the sensor shows excellent reproducibility, ensuring consistent performance across multiple samples and tests, and remarkable long-term stability, which is essential for practical applications in real-world settings. The superiority in the performance compared with reported electrodes, can be attributed to its optimized surface morphology, increased electroactive surface area, and highly efficient charge-transfer arising from the synergistic effect of the incorporated ZnCo_2_O_4_/MWCNTs nanocomposite. Overall, the present work underscores the advantages of material-engineered electrode interfaces and presents a robust, accurate and promising sensing platform for accurate and reliable RF detection.

## Conflicts of interest

There is no conflicts of interest for the publication of the article.

## Supplementary Material

RA-016-D6RA00420B-s001

## Data Availability

The data underlying this study are available in the published article and its supporting information (SI). Supplementary information: Fig. S1: XRD pattern of MWCNT; Fig. S2: EDX mapping images; Fig. S3: interference effects of 10-fold concentrations of AA, UA, glucose, vitamin B_1_ and vitamin B_6_ on the RF response. See DOI: https://doi.org/10.1039/d6ra00420b.
