# Supplementary material for: Carbon nanotube integrated MOF-derived ZnCo2O4: a nanohybrid electrochemical platform for riboflavin sensing
Source: RSC Adv. 2026 Mar 26;16(18):16601–12. doi: 10.1039/d6ra00420b (PMC13019457; doi:10.1039/d6ra00420b)
Supplement: RA-016-D6RA00420B-s001 [file RA-016-D6RA00420B-s001.pdf]

Supplementary Information (SI) for RSC Advances.
This journal is © The Royal Society of Chemistry 2026

Supplementary Information

Carbon Nanotube Integrated MOF-Derived $ZnCo_2O_4$: A Nanohybrid Electrochemical Platform for Riboflavin Sensing

Ankita K. Dhukate[a], Sajid B. Mullani[a,e], Navaj B. Mullani[c], Tukaram D. Dongale[b,d], Sagar D. Delekar[a]*

[a]Department of Chemistry, Shivaji University, Kolhapur, Maharashtra 416004, MS, India.

[b]School of Nanoscience and Biotechnology, Shivaji University, Kolhapur, Maharashtra 416004, MS, India.

[c]School of Physics, Center for Research on Adaptive Nanostructures and Nanodevices, Advanced Material and Bioengineering Research Centers Trinity College, Dublin, Ireland.

[d]Functional Material and Materials Chemistry Laboratory, Department of Physiology, Saveetha Institute of Medical and Technical Sciences, Saveetha University, Chennai 600077, Tamilnadu, MS, India.

[e]Department of Chemistry, Bhogawati Mahavidyalaya, Kurukali, Maharashtra 416001, MS, India.

***Corresponding author:**

Prof. (Dr.) Sagar D. Delekar

E-mail: sddelekar7@rediffmail.com

(Tel.:+91-231-2609100, Fax: +91-231-2692333

1. **XRD pattern of MWCNT**

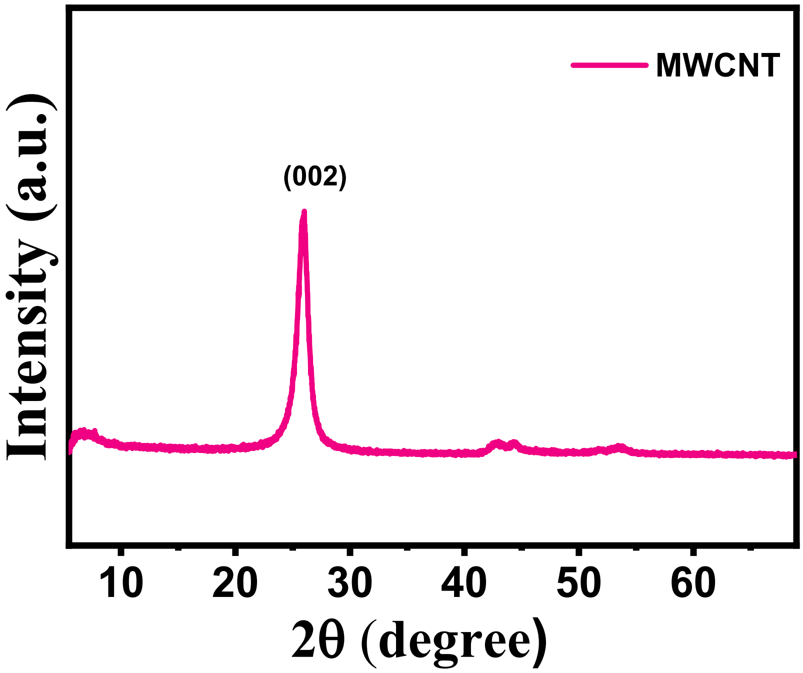

Fig. S1 (a) XRD patterns of MWCNT.

2. **EDX mapping images**

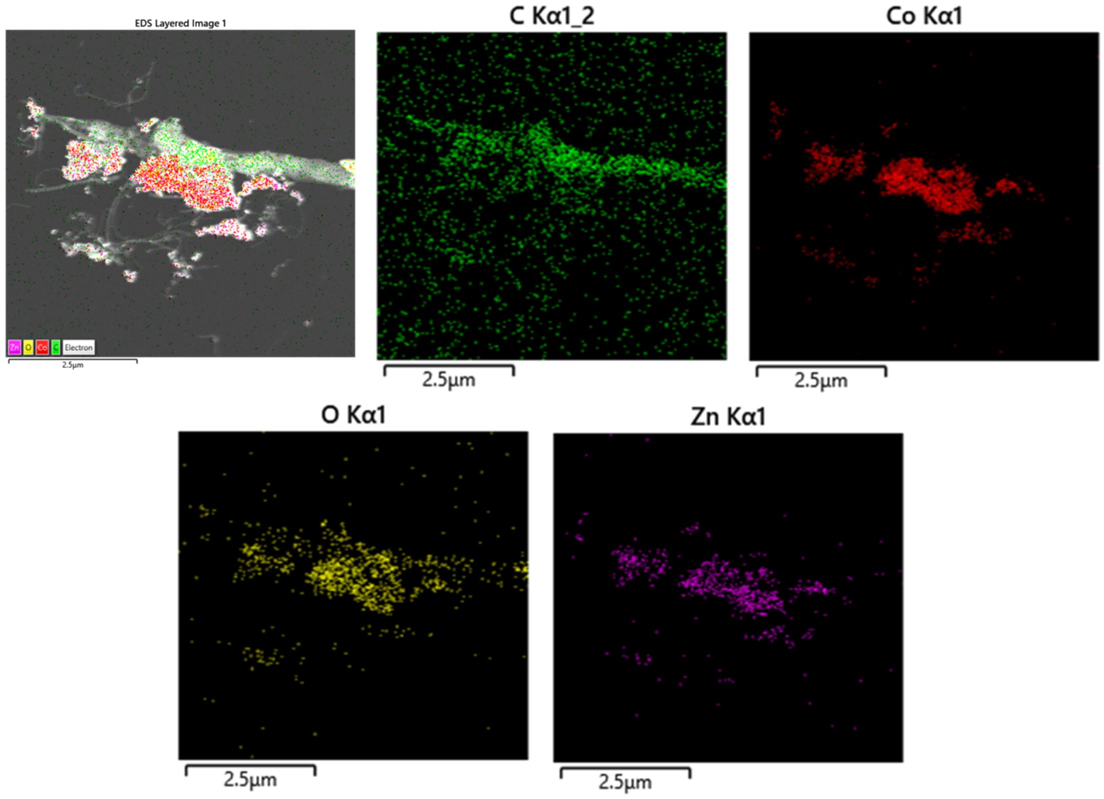

Fig. S2 EDX mapping images of C, Co, O, and Zn

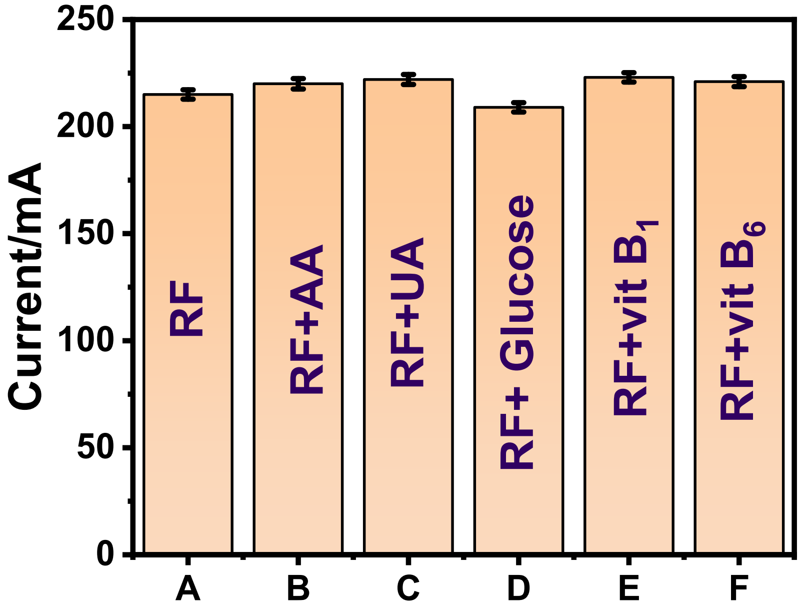

Fig. S3 Interference effects of 10-fold concentrations of AA, UA, glucose, vitamin B_1 and vitamin B_6 on the RF response.
